# Statistical analysis of childhood and early adolescent externalizing behaviors in a middle low income country

**DOI:** 10.1016/j.heliyon.2020.e03377

**Published:** 2020-02-07

**Authors:** Sheila A. Bishop, Hilary I. Okagbue, Jonathan A. Odukoya

**Affiliations:** a Department of Mathematics, Covenant University, Nigeria; b Department of Psychology, Covenant University, Nigeria

**Keywords:** Psychology, Externalizing behaviour, Aggression, Delinquency, Hyperactivity, Questionnaire, Statistics, Nigeria

## Abstract

The article show the pattern of externalizing behavior across age, gender, school type, and school level, with reference to aggression, delinquency, and hyperactivity. The study samples were primary school pupils and secondary school students from three selected Local Government Areas (LGA) in Ogun State, Nigeria [Ado-Odo/Ota, Ifo, and Yewa South]. Their ages ranged from 10 to 20 years. The student/pupil sample was 1770 in all. The instrument used was an adapted version of Achenbach's child behavior checklist and youth self-report. Basic descriptive statistics like frequency, percentage, mean, standard deviation, as well as non-parametric statistics like Phi-coefficient, Chi-square, Goodman and Kruskal's gamma, Mann Whitney U test and Kruskal Wallis H test were utilized. Inferential parametric statistics like Pearson r, analysis of variance and simple regression were also utilized. Four major findings were reported. Firstly, the private schools irrespective of age, gender and level, scored higher than the public school in aggression, delinquency, and hyperactivity. Secondly, aggression is higher in secondary schools, while delinquency and hyperactivity are more prevalent in primary schools. Thirdly, school level and school type are the strongest predictors of externalizing behavior. Lastly, correspondence analysis showed a similar behavioral pattern for the three behaviors and three distinct behavioral patterns. i). Respondents aged 10 and below and those in primary schools (ii). Male, public and between 16 and 20. iii). Private, secondary, female and between 11 and 15. Implications of the study are discussed.

## 1 Introduction

The present study analyzes the data published earlier on the externalizing behavior of primary (elementary) school pupils and secondary (high school) students of three local government areas of Ota in Ogun State, Nigeria [1]. The study is motivated by the quest to obtain the pattern of externalizing behavior (aggression, delinquency, and hyperactivity) using four (4) demographic variables (age, gender, school type, and school level). The study has not been thoroughly considered in a middle-income setting and the demographic variables are yet to be investigated in a single context. Besides, the instrument of data collection is unique, designed to suit the socio-demographics and will be shown later, to be capable of detecting externalizing behavior. Moreover, the results presented in [1] is accentuated to reflect hidden behavioral patterns and deepen our understanding of externalizing behavior.

Behavior is largely a product of thinking. There exist two major classes of behavioral disorders in children and adolescents. Externalizing behaviors are negative behaviors that are channeled towards the external environment while internalizing behavior is directed towards self and may not be disruptive as externalizing behavior [2, 3]. The two major behavioral disorders are inmate in children in active or dormant form. Internalizing behavior can manifest as withdrawal, depression, nervousness, and solitude. Externalizing behavior can manifest as rebellion to constituted authority or failure to comply with stated rules, aggressive tendencies, anti-social behavior, attention deficiencies and disruptive attitudes triggered by impulsivity and under control of emotions. Both behavior disorders differs by their regulatory tactics [4], although they are both influenced by teacher-child conflict [5, 6] and can be as a result of biological processes such as hereditary and genetic [7], ailments [8], prenatal cocaine exposure [9], prenatal maternal stress [10], pregnancy-related complications [11] and shared environment [12]. The combination of genetic and environmental factors have been described to be the major predictors of externalizing behavior [13]. Children's exposure to heavy metal contamination [14] and noise pollution [15] exhibit high levels of deviant and disruptive behaviors. Natural disasters like earthquakes [16] and hurricanes [17] can trigger externalizing tendencies in children and young adults.

Economic problems, poverty [18], unemployment [19], family conflicts or adversity [20], parental mental health [21] and tobacco use [22] are also positively associated with or predictors of both internalizing and externalizing behaviors. Weak social structure and social exclusion are also breeding grounds for behavioral deviations. Exposure to violent media predisposes both children and adults to both internalizing and externalizing behavioral problems [23].

Children of depressed mothers [24] or adolescents that have experienced parental differential treatments [25], children and adults from persistent or concordant drinking parents [26], children that have low receptive language skills [27] and children and adults from polygamous family backgrounds [28] are at a high risk of exhibiting externalizing behavior. Adolescence deviant peer group affiliations [29], adolescents with tattooing and body piercing [30], those having history or profile of jailed parents [31] and those that had remained impervious to behavioral corrections are at a high risk. Adults that engage in absenteeism [32] and truancy in high schools [33] and cyberbullying [34] are most likely to exhibit externalizing behavior.

Childhood and adolescent externalizing behavior is a serious public health issue [35] and a predictor of later life disruptive behavior, violence, substance use and crime [36, 37, 38]. Peer abusers are example of people who have history of childhood externalizing problems [39].

Early researchers classified externalizing behavioral problems as disruptive, aggressive and hyperactive [40]. Although deviant, antisocial, conduct problem and under controlled are some of the terms used in this context [41]. Socioemotional behaviors have been used to describe externalizing and internalizing behaviors [42]. This paper adopted the widely used three externalizing behavior classification or constructs recommended by [43], which are aggression, delinquency, and hyperactivity. The three terms are interrelated and they are scarcely studied together. Nevertheless, they are all known as antisocial and deviant behaviors.

Externalizing behaviors seem to be attributed more to boys [44] while internalizing behaviors are oriented towards girls [45]. Racial and gender differences are often combined in terms of moderation or mediation with other variables and used to establish a relationship with externalizing behavior [46]. Surprisingly, intervention programs targeted at addressing externalizing behavioral disorders are independent of gender [47] and helps to improve school adjustment [48, 49] and overall academic performance of the students or pupils [50, 51]. Disruptive behavior reduces the time for learning and consequently affects the grades [52]. Positive parenting behaviors [53] and an increased number of adult supervision time [54] could be combined with an intervention program to halt the aggravation of externalizing behavior, thereby unblocking the minds of young people and preventing them from rebelling against the acceptable behavior. Regrettably, the use of sporting activities as an intervention program is yet to be found suitable in addressing externalizing behavior whereas active engagement of sporting activities have shown to be useful in tackling internalizing behavioral problems [55]. In the same vein, low socioeconomic status [56] and declining life satisfaction [57] can attenuate the effect of the intervention program.

## 2 Aggression

Aggression consists of physical, emotional and verbal behaviors that are intended to cause harm or injury, hurt or threaten others [58]. Aggression can be directed to children, adults, and animals [59]. Cruelty to pets is an example. The understanding of the concept of aggression takes different forms; it can be viewed as a personality trait, a symptom or a behavioral pattern [60]. The aim may be to protect oneself but in an enormous way or to harm self or others (bullying). In the case of bullying, the victims are often weaker and younger while the aggressors are often stronger, male and older [61]. Researches are yet to determine whether under control of emotions accentuate aggressive behavior in a subconscious or premeditated manner, hence making the classification of aggression difficult. Aggression is resident in the recess of the mind waiting for an external stimulus to trigger it.

Research on aggression has revealed that childhood and adolescent aggression can transmit to adulthood and cause serious antisocial behavioral disorders such as violence, murder, and crime [62], although this has been recently disputed. The authors noted that aggression in children is quite different from an adult as environmental and biological factors can accentuate or attenuate aggressive tendencies over time [63]. This is expected to have far-reaching implications, especially in counseling, learning, intervention, and parenting. Hitherto, childhood aggression continues to predict antisocial behavior in adulthood such as sexual aggression and violence [64].

In terms of gender, boys are generally found to engage in physical and verbal aggression while girls engage in verbal and emotional aggression [65]. Emotional aggression is relative and can take the form of slander, exclusion, neglect and malicious gestures [66]. Some researchers noted that boys are oriented towards direct aggression while the girls are known for indirect aggression [67]. Whichever classification that applies, the issue of aggression is due to biological and psychosocial factors. Alcohol use [68] and substance abuse are some of the risk behaviors that can predict adolescent and adult aggression. Children and young adults who experienced a low level of adequate parenting [69], insensitive and harsh parenting [70] and child to parent violence [71] are most likely to be aggressive. In addition, those that engage in excessive risk-taking tend to be aggressive in adulthood [72].

Regression of academic performance, deteriorating intellectual capabilities, injury and deaths (case of school shootings), loss of property and health issues are some of the consequences of aggressive behavior [73]. The rising global incidence of the manifestations of aggressive behavior among young people warrants urgent intervention programs to stem the ugly tide [74].

## 3 Delinquency

Delinquency can manifest as the following antisocial behaviors; robbery, vandalism, puffery, burglary, theft, mugging, drug or substance use, arson, and violence. Juvenile delinquency is a technical terminology used in this context, to describe a situation where an adolescent below the statutory years of age commits acts that can be imputed as a crime if the person is an adult. At times, the severity of the committed acts can warrant an amendment to crime and as such, the offender or the accused can be treated as an adult [75]. The differences in jurisdiction, culture, race, and political environments forced researchers to adopt the widely used delinquent behaviors listed in [76], which serves as a unified instrument of measuring behavioral disorders, which is independent of jurisdiction and environment. Although modification exists. Some of the antisocial behaviors in these aspects are lying, truancy, peer pressure, and bad company, stealing and cheating [77, 78]. Hence, the concept of delinquency described in this paper is the aspects of antisocial behavior that do not involve violent acts, loosely known as nonviolent delinquency [79]. No matter the definition, delinquency is loosely viewed as behaving outside the parameters of set values. However, delinquent behavior has been proven a predictor of criminal behavior [80] and victimization [81], drunkenness and drug use [82] in adults.

Risk factors of delinquency are parenting style, family alcoholism [83], the influence of siblings [84], peer affiliation or pressure, peer rejection, genetic, poverty [85], and environmental factors. The effect of parenting style on delinquent behavior is the same for males and females [86], surprisingly, parental monitoring does not affect the delinquency behavior in boys [87]. Moreover, no gender difference was observed for delinquent behavior [88, 89], which is contrary to the findings of [90] that attributed high susceptibility to boys. Exposure to violence [91] and personal identity formation [92] have been implicated in predicting delinquent behaviors in young people. For young females, intimate violence can trigger delinquent episodes [93]. Students with disabilities have been identified to have more propensity to be suspended in school due to their delinquent behaviors [94].

Researchers have categorized violent antisocial behaviors like aggression and non-violent ones as delinquency, although, an inter-lapping exists between them making distinct classification a herculean task. For example, handgun carrying is both a delinquent and aggressive behavior, the intention and mode of use, notwithstanding [95]. The presence of aggressive behavior is likely to trigger delinquent behavior and vice versa [96], for instance, peer delinquency is associated with perceived relational aggression in early adolescence [97].

## 4 Hyperactivity

Generally, hyperactivity refers to two major types of behavioral disorders, namely, restlessness (impulsivity) and inattention (attention deficits). Attention-deficit/hyperactivity disorder (ADHD) is the term commonly used in the literature [98]. Children and early adolescents can manifest any of the two behaviors; hence, it is cumbersome to isolate inattentiveness from hyperactivity-impulsivity. Inattentiveness includes behaviors such as limited attention span, easily distracted, disorientation, and inability to maintain, manage and organize given tasks, difficulty to listen and carry out given instructions, forgetfulness and making excessive mistakes in a given task. Hyperactivity-impulsivity includes behaviors such as inability to sit still, having little or no sense of danger, risk-taking, interrupting discussions in class or conversations, unthoughtful acts, excessive talking and physical movement, difficulty in queuing and difficulty keeping quiet. Besides, abnormally excitable is typical for hyperactive children and young adults. This article adopts hyperactivity to describe the two behaviors.

The diagnosis of hyperactivity is key to proper treatment and counseling. Diagnosis accuracy reduces with decreasing age; because any child can exhibit hyperactivity [99]. Clinical psychologists treat hyperactivity as a neurodevelopmental disorder [100], which can be identified by the use of electroencephalography (EEG), combined with convolutional neural networks [101]. Maternal health during pregnancy [102] and prenatal exposure to insecticides [103] are associated with hyperactivity. Children diagnosed with hyperactivity have been found to some extent, to experience written expression difficulties [104].

Hyperactivity is more prevalent in children [105] and more evident in boys than girls [106], but the manifestation is different as personality is independent of gender. The high rate of prevalence of hyperactive behavior in children of tender age was reechoed in a study conducted in a low middle-income country [107]. Nonetheless, hyperactivity is implicated in crime [108], road accidents [109] and violence [110] in adults.

Parenting is one key variable that predicts hyperactivity [111]. Negative parenting such as excessive punishment, parenting isolation, violent and abusive parents, gambling parents, alcoholism and attention dispersion can alter the character of young children and push them into antisocial behavior [112]. The negative familial variables that predict hyperactivity in young people could be exacerbated by excruciating poverty, homelessness, and destitution [113]. The onset of puberty can trigger hyperactivity in particular and externalizing behavior in general [114]. Mind-wandering is associated with hyperactivity and impairment [115]. This is expected since impairment in inhibitory control is one of the defining characteristics of hyperactivity [116]. Dietary habits which include excessive eating [117, 118] and unhealthy eating patterns [119] and high levels of sedentary behavior [120] are significantly associated with hyperactive behavior.

## 5 Study population

The study population was primary school pupils and secondary school students of different schools in three (3) selected Local Government Areas (LGA) of Ogun State, Nigeria. The local government areas are Ado-Odo/Ota, Ifo, and Yewa South. They were chosen because of proximity and similar demographics. The total population of the school were not obtained because in some schools, the school management refused to divulge the information while in some schools, the management gave the condition that their teachers are the only one that are authorized to administer the questionnaires. Parental consent were proxy as the parents consented that only the teachers can administer the questionnaires for health and security reasons.

The demographic variables are a). school type (public, private), b). age group (10 and below, 11–15, 16–20), c). gender (female, male), and d). school level (primary, secondary). School type implies privately owned or publicly funded.

## 6 Instrument of data collection

A Likert scale Questionnaire was obtained from the modifications of Achenbach's manual for child behavior checklist and manual for the youth self-report. The idea is to design a questionnaire that is suited for the studied demographics. The questionnaire consists of 100 questions (variables). Only three responses were available and coded 0, 1 and 2. The highest obtainable score is two hundred (200) and the least is zero (0). High scores imply high externalizing behavior. The details can be seen in [1].

## 7 Survey methodology

Cluster sampling was used to select the schools in the respective LGAs. Thereafter, simple random sampling was used to draw the samples. Parental consent was sought through the respective school administrators. All the schools are duly registered and licensed to admit students and or pupils. The questionnaire was written in simple English words for easy comprehension. The school teachers were briefly trained and assisted in the administration of the questionnaires.

## 8 Demographics analysis

The survey was carried out between November and December 2016. Two thousand (2000) questionnaires were distributed and 1770 were finally analyzed. A detailed analysis can be found in [1].

## 9 Analysis of total externalizing behavior scores

The total scores obtained from the analysis of the questionnaires are the measure of the externalizing behavior presented in [1]. The present study dissects the total scores into three behavioral components and the results are discussed. P-value < 0.05 is considered significant.

## 10 Contingency analysis

Contingency analysis is often applied in psychological studies especially in the analysis of responses from structured or scaled questionnaires. Cross tabulation was used to classify the data and the Chi-square Pearson test was applied subsequently to obtain the association or independence of the demographic factors and the externalizing behavior variables which can be interpreted with the aid of p-values. Significance implies association. Correlation among the categorical variables was shown using Phi-coefficient (mean square contingency coefficient) and Goodman and Kruskal's gamma simply known as gamma. The contingency analysis is presented for examining the association between externalizing behavior scores (the measure of externalizing behavior) and school type (Table 1), age group (Table 2), gender (Table 3) and school level (Table 4).

**Table 1:** Summary of the contingency analysis between the respondents’ school type and total externalizing behavior score.

Test	Value
Pearson's Chi-square	205.764743***
Phi	0.340956***
Goodman and Kruskal's gamma	0.177257***
Pearson's R	0.139328***

***p < 0.001.

**Table 2:** Summary of the contingency analysis between the respondents’ age group and total externalizing behavior score.

Test	Value
Pearson's Chi-square	308.401651
Phi	0.417419
Goodman and Kruskal's gamma	-0.035409
Pearson's R	-0.024815

**Table 3:** Summary of the contingency analysis between the respondents’ gender and total externalizing behavior score.

Test	Value
Pearson's Chi-square	183.958027*
Phi	0.322383*
Goodman and Kruskal's gamma	-0.059198*
Pearson's R	-0.055951**

*p < 0.05, **p < 0.01.

**Table 4:** Summary of the contingency analysis between the respondent's school level and total externalizing behavior score.

Test	Value
Pearson's Chi-square	216.363803***
Phi	0.349628***
Goodman and Kruskal's gamma	-0.060586
Pearson's R	-0.040193

***p < 0.001.

The results of the contingency analysis presented in Tables 1, 2, 3, and 4 showed that externalizing behavior is associated with school type, gender, and school level. However, no association exists between externalizing behavior and age.

Also, contingency analysis was performed to determine the association between the 100 externalizing behavior variables and the demographic variables and the outcomes are presented for school type (Table 5), age group (Table 6), gender (Table 7) and school level (Table 8). The summary of all the significant associations between the demographic and 100 externalizing behavior variables is presented in Table 9.

**Table 5:** Summary of the contingency analysis between the respondents’ school type and 100 externalizing behavior variables.

Variable	Chi-square	p-value	Phi	Gamma	Variable	Chi-square	p-value	Phi	Gamma
1	2.313	0.314559	0.036	-0.013	51	31.673	<0.0001	0.134	-0.228
2	11.319	0.003484	0.08	0.135	52	5.118	0.077383	0.054	0.094
3	2.756	0.252037	0.039	0.057	53	23.741	0.000007	0.116	0.197
4	1.245	0.536531	0.027	0.07	54	20.636	0.000033	0.108	0.183
5	15.865	0.000359	0.095	0.159	55	110.003	<0.0001	0.249	0.488
6	2.066	0.355927	0.034	0.043	56	15.471	0.000437	0.093	0.198
7	33.345	<0.0001	0.137	0.195	57	17.998	0.000124	0.101	0.172
8	2.346	0.309469	0.036	0.04	58	1.19	0.55145	0.026	0.04
9	15.353	0.000464	0.093	-0.147	59	8.334	0.015501	0.069	0.086
10	18.329	0.000105	0.102	0.191	60	43.807	<0.0001	0.157	-0.261
11	6.203	0.044991	0.059	0.085	61	41.202	<0.0001	0.153	0.258
12	6.51	0.038574	0.061	-0.039	62	5.573	0.061646	0.056	0.043
13	1.255	0.53391	0.027	-0.057	63	6.991	0.03033	0.063	0.062
14	0.942	0.624465	0.023	-0.018	64	19.336	0.000063	0.105	0.161
15	2.999	0.223194	0.041	0.07	65	3.586	0.166421	0.045	0.076
16	14.928	0.000573	0.092	0.13	66	19.674	0.000053	0.105	0.181
17	21.137	0.000026	0.109	0.186	67	1.303	0.52136	0.027	0.021
18	10.447	0.005388	0.077	0.141	68	21.18	0.000025	0.109	0.197
19	2.0424	0.360168	0.034	-0.064	69	8.848	0.011989	0.071	0.119
20	6.926	0.031337	0.063	0.085	70	5.46	0.06523	0.056	0.094
21	4.654	0.097587	0.051	0.089	71	8.525	0.014086	0.069	0.126
22	5.484	0.064425	0.056	-0.084	72	15.1	0.000526	0.092	0.099
23	3.21	0.200875	0.043	0.041	73	11.963	0.002525	0.082	0.14
24	19.75	0.000051	0.106	0.127	74	1.218	0.543825	0.026	0.045
25	30.791	<0.0001	0.132	-0.218	75	7.031	0.029733	0.063	0.092
26	58.082	<0.0001	0.181	0.322	76	1.805	0.405559	0.032	0.058
27	5.606	0.060637	0.056	-0.06	77	2.418	0.298436	0.037	-0.064
28	2.976	0.225823	0.041	0.069	78	40.075	<0.0001	0.151	0.24
29	13.189	0.001368	0.086	0.079	79	54.595	<0.0001	0.176	0.328
30	8.689	0.012979	0.07	0.118	80	2.375	0.305006	0.037	0.098
31	13.802	0.001007	0.088	0.076	81	37.694	<0.0001	0.146	0.227
32	37.725	<0.0001	0.146	0.257	82	2.5	0.286491	0.038	0.065
33	10.573	0.005059	0.077	0.1494	83	10.212	0.006061	0.076	0.131
34	15.7	0.00039	0.094	0.137	84	0.7077	0.701971	0.02	0.034
35	0.508	0.775675	0.017	0.025	85	14.218	0.000818	0.09	0.151
36	0.521	0.770805	0.017	-0.007	86	33.204	<0.0001	0.137	0.25
37	3.071	0.21533	0.042	-0.079	87	16.243	0.000297	0.096	0.162
38	19.063	0.000073	0.104	0.052	88	19.723	0.000052	0.106	0.179
39	12.42	0.002009	0.084	0.054	89	27.102	0.000001	0.124	0.219
40	5.596	0.060931	0.056	0.096	90	19.903	0.000048	0.106	0.184
41	4.677	0.096451	0.051	0.086	91	13.922	0.000948	0.089	0.148
42	29.325	<0.0001	0.129	0.23	92	17.462	0.000161	0.099	0.175
43	21.026	0.000027	0.109	0.19	93	32.323	<0.0001	0.135	0.24
44	61.939	<0.0001	0.187	0.301	94	3.453	0.177928	0.044	0.071
45	63.876	<0.0001	0.19	0.346	95	10.672	0.004814	0.077	0.131
46	7.482	0.023732	0.065	0.162	96	11.944	0.002549	0.082	0.142
47	3.658	0.160588	0.045	-0.046	97	0.841	0.656883	0.022	-0.04
48	3.037	0.219054	0.041	0.038	98	1.476	0.478128	0.029	-0.01
49	0.848	0.654422	0.022	0.036	99	60.596	<0.0001	0.185	0.392
50	0.46	0.794571	0.016	0.017	100	8.264	0.01605	0.068	0.112

**Table 6:** Summary of the contingency analysis between the respondents’ age group and 100 externalizing behavior variables.

Variable	Chi-square	p-value	Phi	Gamma	Variable	Chi-square	p-value	Phi	Gamma
1	16.164	0.002807	0.096	0.067	51	12.558	0.01365	0.084	0.082
2	20.039	0.000491	0.106	-0.003	52	37.163	<0.0001	0.145	0.069
3	39.421	<0.0001	0.149	-0.189	53	13.198	0.010346	0.086	-0.099
4	11.541	0.021112	0.081	-0.154	54	13.052	0.011024	0.086	0.009
5	48.414	<0.0001	0.165	-0.185	55	15.024	0.004652	0.092	-0.105
6	26.368	0.000027	0.122	-0.201	56	24.613	0.00006	0.118	-0.224
7	22.377	0.000169	0.112	-0.119	57	9.936	0.041512	0.075	-0.093
8	15.919	0.00313	0.095	-0.026	58	76.245	<0.0001	0.208	0.268
9	9.631	0.047117	0.074	0.094	59	7.276	0.121989	0.064	0.023
10	33.048	0.000001	0.137	0.126	60	54.491	<0.0001	0.175	0.191
11	7.348	0.11858	0.064	0.132	61	2.188	0.701289	0.035	0.04
12	6.155	0.187845	0.059	-0.122	62	8.527	0.074062	0.069	0.046
13	11.131	0.025127	0.079	0.003	63	17.546	0.001513	0.1	0.129
14	21.884	0.000211	0.111	-0.125	64	10.781	0.02914	0.078	0.103
15	1.611	0.806849	0.03	0.017	65	4.005	0.405393	0.048	-0.013
16	11.114	0.025311	0.079	-0.011	66	8.949	0.062381	0.071	-0.1
17	12.89	0.011827	0.085	-0.04	67	13.338	0.009738	0.087	-0.076
18	18.639	0.000925	0.103	-0.075	68	17.768	0.00137	0.1	-0.087
19	22.265	0.000177	`0.112	0.051	69	21.094	0.000303	0.109	-0.049
20	4.767	0.312028	0.052	0.008	70	2.698	0.609652	0.039	0.01
21	13.757	0.008111	0.088	0.053	71	7.215	0.124976	0.064	-0.003
22	40.342	<0.0001	0.151	0.245	72	6.675	0.15409	0.061	0.043
23	11.156	0.024869	0.079	-0.035	73	8.889	0.063941	0.071	-0.005
24	8.117	0.087372	0.068	-0.031	74	16.069	0.002928	0.095	-0.033
25	39.86	<0.0001	0.15	0.165	75	9.126	0.058029	0.072	0.067
26	16.407	0.002519	0.096	-0.07	76	7.682	0.103933	0.066	-0.149
27	46.066	<0.0001	0.161	-0.193	77	15.061	0.004576	0.092	-0.049
28	16.309	0.002631	0.096	-0.037	78	14.172	0.006767	0.089	-0.123
29	3.047	0.549971	0.041	-0.053	79	12.375	0.01477	0.084	-0.132
30	17.322	0.001674	0.099	0.111	80	7.787	0.09969	0.066	-0.111
31	2.851	0.583067	0.04	0.018	81	14.15	0.00683	0.089	0.003
32	6.289	0.178581	0.06	-0.054	82	12.522	0.013861	0.084	0.009
33	7.86	0.096825	0.067	0.05	83	58.662	<0.0001	0.182	-0.251
34	4.42	0.352142	0.05	0.03	84	16.286	0.002658	0.096	-0.044
35	26.995	0.00002	0.123	0.131	85	17.148	0.001809	0.098	0.084
36	11.215	0.024246	0.08	0.054	86	2.24	0.691699	0.036	-0.006
37	5.794	0.215035	0.057	0.035	87	7.241	0.12371	0.064	0.008
38	31.951	0.000002	0.134	-0.076	88	9.264	0.054831	0.072	0.031
39	26.306	0.000027	0.122	-0.146	89	10.319	0.035379	0.076	0.067
40	22.204	0.000183	0.112	-0.122	90	38.658	<0.0001	0.148	0.168
41	20.702	0.000363	0.108	0.1	91	6.677	0.153977	0.061	-0.039
42	7.471	0.112995	0.065	-0.044	92	14.869	0.00498	0.092	-0.068
43	3.577	0.46629	0.045	-0.061	93	24.837	0.000054	0.118	-0.168
44	12.783	0.012388	0.085	-0.164	94	0.642	0.958267	0.019	0.011
45	38.368	<0.0001	0.147	-0.231	95	6.553	0.161475	0.061	0.041
46	1.111	0.892475	0.025	0.021	96	5.132	0.273995	0.054	-0.067
47	6.428	0.169358	0.06	0	97	10.748	0.029548	0.078	-0.13
48	8.93	0.062884	0.071	0.018	98	6.676	0.154057	0.061	-0.078
49	8.317	0.080637	0.069	0.071	99	18.086	0.001187	0.101	-0.174
50	19.897	0.000523	0.106	0.035	100	22.471	0.000161	0.113	-0.116

**Table 7:** Summary of the contingency analysis between the respondents’ gender and 100 externalizing behavior variables.

Variable	Chi-Square	p-value	Phi	Gamma	Variable	Chi-Square	p-value	Phi	Gamma
1	1.796	0.407368	0.032	0.046	51	7.642	0.021906	0.066	-0.092
2	5.913	0.051986	0.058	-0.094	52	4.458	0.107623	0.05	-0.065
3	2.295	0.317441	0.036	-0.023	53	0.696	0.705987	0.02	-0.031
4	0.382	0.826244	0.015	0.005	54	6.865	0.032298	0.062	0.102
5	1.495	0.473595	0.029	0.02	55	7.342	0.025447	0.064	-0.094
6	38.463	<0.0001	0.147	0.268	56	13.102	0.001429	0.086	-0.156
7	2.406	0.300271	0.037	-0.049	57	1.822	0.402137	0.032	-0.054
8	1.128	0.56881	0.025	0.034	58	4.492	0.105809	0.05	-0.088
9	3.521	0.171941	0.045	0.077	59	10.302	0.005792	0.076	0.11
10	1.601	0.449019	0.03	-0.047	60	3.697	0.157461	0.046	0.075
11	7.844	0.0198	0.067	-0.172	61	0.17	0.91868	0.01	-0.003
12	1.444	0.485819	0.029	-0.081	62	3.988	0.13617	0.047	-0.059
13	1.521	0.467416	0.029	-0.06	63	0.54	0.763254	0.017	-0.02
14	3.558	0.168783	0.045	-0.045	64	3.794	0.15003	0.046	-0.055
15	5.27	0.07173	0.055	0.09	65	6.813	0.033157	0.062	0.074
16	17.345	0.000171	0.099	-0.113	66	7.69	0.021386	0.066	-0.103
17	1.907	0.385296	0.033	-0.053	67	7.735	0.020907	0.066	-0.129
18	1.927	0.381552	0.033	-0.031	68	11.565	0.003081	0.081	-0.148
19	6.675	0.035522	0.061	0.107	69	34.889	<0.0001	0.14	-0.227
20	1.539	0.46322	0.029	-0.04	70	0.338	0.844603	0.014	0.022
21	0.303	0.859303	0.013	0.022	71	1.623	0.444104	0.03	-0.053
22	19.555	0.000057	0.105	0.179	72	27.536	0.000001	0.125	-0.265
23	7.83	0.019936	0.067	-0.117	73	2.57	0.276673	0.038	-0.063
24	5.22	0.073539	0.054	-0.095	74	0.193	0.908045	0.01	-0.005
25	5.991	0.050012	0.058	-0.098	75	2.297	0.317047	0.036	-0.036
26	49.037	<0.0001	0.166	-0.292	76	5.547	0.062453	0.056	-0.124
27	8.55	0.013914	0.07	-0.11	77	3.153	0.206697	0.042	-0.033
28	6.517	0.038453	0.061	-0.099	78	3.645	0.161584	0.045	-0.065
29	7.148	0.028037	0.064	-0.098	79	11.22	0.00366	0.08	-0.151
30	1.853	0.396009	0.032	0.008	80	27.296	0.000001	0.124	-0.317
31	4.827	0.089517	0.052	0.084	81	8.087	0.017537	0.068	-0.112
32	5.939	0.051325	0.058	-0.037	82	1.078	0.583186	0.025	-0.028
33	7.461	0.02398	0.065	0.12	83	4.534	0.103612	0.051	0.065
34	0.149	0.928297	0.009	0.014	84	5.457	0.065334	0.056	-0.112
35	0.893	0.639837	0.022	0.04	85	0.059	0.970988	0.006	0.009
36	0.858	0.651242	0.022	-0.001	86	21.205	0.000025	0.109	-0.193
37	10.731	0.004675	0.078	0.141	87	4.778	0.091723	0.052	0.083
38	2.862	0.239011	0.04	-0.005	88	0.128	0.937939	0.009	0.008
39	6.681	0.035422	0.061	-0.098	89	16.068	0.000324	0.095	-0.161
40	2.017	0.364693	0.034	-0.055	90	30.512	<0.0001	0.131	-0.217
41	9.806	0.007425	0.074	0.121	91	10.412	0.005484	0.077	0.105
42	7.52	0.023279	0.065	-0.101	92	0.465	0.792668	0.016	0.005
43	8.893	0.011721	0.071	-0.132	93	22.845	0.000011	0.114	-0.203
44	4.433	0.108988	0.05	-0.092	94	3.005	0.222575	0.041	0.058
45	11.422	0.00331	0.08	-0.15	95	1.143	0.564783	0.025	-0.029
46	16.388	0.000276	0.096	-0.247	96	2.293	0.317772	0.036	-0.063
47	0.118	0.942601	0.008	-0.005	97	2.349	0.308984	0.036	0.006
48	3.651	0.161168	0.045	0.045	98	2.041	0.360389	0.034	-0.019
49	0.576	0.749735	0.018	-0.025	99	1.819	0.40267	0.032	-0.052
50	1.725	0.422105	0.031	0	100	5.139	0.076565	0.054	-0.079

**Table 8:** Summary of the contingency analysis between the respondents’ school level and 100 externalizing behavior variables.

Variable	Chi-square	p-value	Phi	Gamma	Variable	Chi-square	p-value	Phi	Gamma
1	40.472	<0.0001	0.151	0.029	51	35.887	<0.0001	0.142	-0.271
2	5.753	0.056343	0.057	0.108	52	105.476	<0.0001	0.244	0.34
3	105.805	<0.0001	0.244	-0.469	53	4.506	0.105078	0.05	0.004
4	26.689	0.000002	0.123	-0.338	54	26.925	0.000001	0.123	0.205
5	32.917	<0.0001	0.136	-0.211	55	2.178	0.336634	0.035	-0.004
6	42.147	<0.0001	0.154	-0.317	56	37.617	<0.0001	0.146	-0.35
7	1.554	0.459882	0.03	0.057	57	2.601	0.272445	0.038	0.006
8	7.849	0.019755	0.067	-0.061	58	93.52	<0.0001	0.23	0.43
9	6.265	0.043607	0.059	0.029	59	51.657	<0.0001	0.171	0.125
10	17.637	0.000148	0.1	0.218	60	67.94	<0.0001	0.196	0.178
11	13.083	0.001442	0.086	-0.098	61	3.428	0.180144	0.044	0.037
12	40.176	<0.0001	0.151	-0.403	62	24.884	0.000004	0.119	0.119
13	7.061	0.029285	0.063	-0.097	63	16.598	0.000249	0.097	0.189
14	39.495	<0.0001	0.149	-0.298	64	17.652	0.000147	0.1	0.164
15	11.174	0.003747	0.079	0.149	65	27.235	0.000001	0.124	0.161
16	27.968	0.000001	0.126	-0.013	66	4.309	0.115937	0.049	-0.097
17	31.572	<0.0001	0.134	-0.181	67	55.144	<0.0001	0.177	-0.384
18	29.455	<0.0001	0.129	-0.124	68	16.649	0.000242	0.097	-0.126
19	30.28	<0.0001	0.131	0.164	69	25.89	0.000002	0.121	-0.043
20	23.221	0.000009	0.115	0.164	70	30.211	<0.0001	0.131	0.239
21	27.204	0.000001	0.124	0.155	71	15.239	0.000491	0.093	0
22	18.095	0.000118	0.101	0.218	72	6.058	0.048374	0.059	-0.04
23	10.066	0.006518	0.075	-0.069	73	30.659	<0.0001	0.132	-0.078
24	10.541	0.005141	0.077	-0.018	74	20.961	0.000028	0.109	-0.124
25	51.399	<0.0001	0.17	-0.027	75	37.581	<0.0001	0.146	0.038
26	35.058	<0.0001	0.141	0.132	76	15.731	0.000384	0.094	-0.283
27	79.167	<0.0001	0.211	-0.343	77	30.196	<0.0001	0.131	-0.165
28	13.751	0.001033	0.088	0.054	78	11.447	0.003268	0.08	-0.012
29	11.685	0.002901	0.081	-0.141	79	10.024	0.006658	0.075	-0.169
30	22.186	0.000015	0.112	0.2	80	22.754	0.000011	0.113	-0.316
31	16.707	0.000236	0.097	-0.102	81	0.176	0.915863	0.01	-0.018
32	10.631	0.004915	0.077	-0.129	82	10.111	0.006373	0.076	-0.097
33	30.046	<0.0001	0.13	0.142	83	49.67	<0.0001	0.168	-0.309
34	13.37	0.00125	0.087	0.048	84	41.637	<0.0001	0.153	-0.342
35	76.139	<0.0001	0.207	0.333	85	5.028	0.080926	0.053	0.105
36	24.724	0.000004	0.118	0.055	86	1.069	0.585969	0.025	-0.055
37	18.244	0.000109	0.102	-0.122	87	11.346	0.003438	0.08	0.149
38	38.042	<0.0001	0.147	-0.231	88	24.404	0.000005	0.117	0.04
39	47.362	<0.0001	0.164	-0.31	89	74.133	<0.0001	0.205	0.328
40	37.802	<0.0001	0.146	-0.175	90	107.167	<0.0001	0.246	0.436
41	54.714	<0.0001	0.176	0.312	91	5.4	0.067222	0.055	-0.036
42	6.323	0.04237	0.06	-0.13	92	14.367	0.000759	0.09	-0.149
43	11.788	0.002756	0.082	-0.099	93	18.43	0.0001	0.102	-0.204
44	65.631	<0.0001	0.193	-0.308	94	11.918	0.002583	0.082	-0.014
45	78.6	<0.0001	0.211	-0.367	95	4.366	0.11273	0.05	0.08
46	10.726	0.004686	0.078	-0.223	96	15.01	0.00055	0.092	-0.186
47	5.008	0.081776	0.053	-0.107	97	22.194	0.000015	0.112	-0.186
48	10.482	0.005295	0.077	-0.071	98	39.153	<0.0001	0.149	-0.29
49	16.156	0.00031	0.096	0.129	99	16.686	0.000238	0.097	-0.234
50	74.075	<0.0001	0.205	0.325	100	54.335	<0.0001	0.175	-0.126

**Table 9:** Significant associations between the demographic and 100 externalizing behavior variables.

Variable	School Type	Age Group	Gender	School Level	Variable	School Type	Age Group	Gender	School Level
1		•		•	51	•	•	•	•
2	•	•			52		•		•
3		•		•	53	•	•		
4		•		•	54	•	•	•	•
5	•	•		•	55	•	•	•	
6		•	•	•	56	•	•	•	•
7	•	•			57	•	•		
8		•		•	58		•		•
9	•	•		•	59	•		•	•
10	•	•		•	60	•	•		•
11	•		•	•	61	•			
12	•			•	62				•
13		•		•	63	•	•		•
14		•		•	64	•	•		•
15				•	65			•	•
16	•	•	•	•	66	•		•	
17	•	•		•	67		•	•	•
18	•	•		•	68	•	•	•	•
19		•	•	•	69	•	•	•	•
20	•			•	70				•
21		•		•	71	•			•
22		•	•	•	72	•		•	•
23		•	•	•	73	•			•
24	•			•	74		•		•
25	•	•		•	75	•			•
26	•	•	•	•	76				•
27		•	•	•	77		•		•
28		•	•	•	78	•	•		•
29	•		•	•	79	•	•	•	•
30	•	•		•	80			•	•
31	•			•	81	•	•	•	
32	•			•	82		•		•
33	•		•	•	83	•	•		•
34	•			•	84		•		•
35		•		•	85	•	•		
36		•		•	86	•		•	
37			•	•	87	•			•
38	•	•		•	88	•			•
39	•	•	•	•	89	•	•	•	•
40		•		•	90	•	•	•	•
41		•	•	•	91	•		•	
42	•		•	•	92	•	•		•
43	•		•	•	93	•	•	•	•
44	•	•		•	94				•
45	•	•	•	•	95	•			
46	•		•	•	96	•			•
47					97		•		•
48				•	98				•
49				•	99	•	•		•
50		•		•	100	•	•		•

The results of the contingency analysis presented in Tables 5, 6, 7, 8, and 9, shows the following: Sixty-two (62), sixty-three (63), thirty-seven (37) and eighty-seven (87) externalizing variables are associated with school type, age, gender, and school level respectively.

## 11 Mean rank of the externalizing behavior variables

The mean rank was done to quantify and rank the variables. The mean rank revealed the pattern of the scores as responded by the pupils and students. The variables with high mean are the most common externalizing behavior exhibited by the respondents. This is presented in Table 10.

**Table 10:** Mean rank of the externalizing variables arranged in descending order.

Variable	Rank	Variable	Rank	Variable	Rank	Variable	Rank	Variable	Rank
Q19	74.51	Q28	59.45	Q82	53.70	Q5	46.49	Q32	40.79
Q50	73.65	Q2	59.20	Q77	53.30	Q71	45.69	Q10	40.68
Q33	73.14	Q7	58.81	Q100	53.24	Q23	45.45	Q44	39.92
Q41	71.49	Q59	58.81	Q34	53.13	Q37	45.30	Q79	39.87
Q35	70.10	Q91	58.12	Q51	53.13	Q24	45.27	Q67	38.10
Q1	69.30	Q30	57.91	Q81	52.41	Q96	45.06	Q13	38.01
Q58	69.01	Q40	57.90	Q27	52.33	Q97	44.98	Q63	37.93
Q70	66.59	Q88	57.25	Q39	51.32	Q61	44.75	Q42	37.50
Q9	66.14	Q49	56.74	Q66	51.16	Q22	44.50	Q84	37.43
Q89	65.95	Q87	56.53	Q48	50.8	Q31	44.41	Q98	37.36
Q65	64.79	Q95	56.40	Q21	50.74	Q26	44.04	Q72	36.28
Q52	63.26	Q62	56.04	Q17	49.77	Q18	43.91	Q55	36.05
Q94	63.24	Q78	55.23	Q25	49.73	Q86	43.55	Q56	34.93
Q90	62.82	Q8	54.58	Q38	49.24	Q6	43.42	Q99	34.75
Q57	61.25	Q69	54.47	Q73	48.85	Q93	42.83	Q80	32.12
Q54	60.11	Q75	54.21	Q64	48.11	Q14	42.59	Q4	32.06
Q20	60.00	Q47	54.16	Q16	47.81	Q3	42.23	Q46	31.93
Q60	59.81	Q53	53.93	Q74	47.76	Q43	41.68	Q11	31.34
Q36	59.55	Q85	53.93	Q92	46.95	Q45	41.62	Q76	31.12
Q15	59.49	Q29	53.76	Q83	46.94	Q68	41.36	Q12	30.64

One of the key objective of this paper is to investigate whether the behavioral differences can be explained by the school level (primary or secondary). The total mean score can computed separately for the school levels and the result is shown in Table 11 where it can be clearly seen that the response from the pupils differs quantitatively from the students. A clear deviation is observed in Tables 10 and 11. This is an indication that the behavioral patterns at school levels differs from the general behavioral which to some extent has proved that behavioral segments differs from the total behavioral pattern.

**Table 11:** The mean score based on the school level.

V	Pr	Se	V	Pr	Se	V	Pr	Se	V	Pr	Se
Q19	65	68	Q30	26	29	Q21	55	40	Q14	14	8
Q50	48	53	Q40	54	37	Q17	43	62	Q3	50	41
Q33	46	22	Q88	50	53	Q25	44	44	Q43	46	45
Q41	18	10	Q49	51	43	Q38	45	56	Q45	28	21
Q35	41	30	Q87	42	52	Q73	18	17	Q68	18	10
Q1	39	25	Q95	34	28	Q64	25	13	Q32	43	42
Q58	48	51	Q62	29	23	Q16	55	55	Q10	48	43
Q70	48	44	Q78	69	76	Q74	50	73	Q44	45	30
Q9	62	64	Q8	41	43	Q92	46	52	Q79	30	17
Q89	18	25	Q69	56	73	Q83	45	55	Q67	40	45
Q65	12	9	Q75	50	53	Q5	29	30	Q13	30	28
Q52	16	7	Q47	37	30	Q71	43	48	Q63	41	49
Q94	23	19	Q53	49	36	Q23	16	21	Q42	47	49
Q90	38	24	Q85	55	38	Q37	30	36	Q84	49	67
Q57	46	54	Q29	57	48	Q24	54	62	Q98	39	63
Q54	36	34	Q82	60	76	Q96	43	39	Q72	51	50
Q20	45	35	Q77	23	18	Q97	35	18	Q55	39	32
Q60	34	27	Q100	29	24	Q61	30	24	Q56	34	25
Q36	71	78	Q34	35	20	Q22	47	44	Q99	58	58
Q15	46	55	Q51	40	22	Q31	54	67	Q80	44	48
Q28	33	39	Q81	15	10	Q26	30	32	Q4	40	29
Q2	23	32	Q27	48	43	Q18	20	17	Q46	37	28
Q7	34	30	Q39	42	38	Q86	39	34	Q11	28	17
Q59	32	30	Q66	43	49	Q6	40	33	Q76	21	14
Q91	39	36	Q48	63	79	Q93	43	44	Q12	48	41

V = variable, Pr = primary, Se = secondary.

The summary of the mean score differences are presented in Table 12 where it can be seen that the primary school pupils have more score than the secondary school students in 58 variables (questions), ties in only 3 variables and the students scored more than the pupils in 39 variables. This is a strong evidence of the externalizing behavior differs by the school level. This will present a useful guide for behavioral intervention and counselling where areas of high mean scores can be investigated and addressed.

**Table 12:** The frequency of the difference between the mean scores of the variables based on the school level.

Difference	Frequency
Primary > Secondary	58
Primary = Secondary	3
Primary < Secondary	39

## 12 Classification of the externalizing variables

The analysis of the externalizing variable was made more meaningful by splitting the 100 variables into aggression, delinquency and hyperactivity showed in Table 13. Independent psychologist carried out the classification and grouped 19 variables under aggression, 57 under delinquency, 18 under hyperactivity and 6 variables were excluded because they cannot be explicitly classified into any of the three behaviors. Overlapping was cited as the reason. Thereafter, the three broad externalizing variables were analyzed.

**Table 13:** Summary of the Classification of the 100 externalizing variables.

Behavior	Variables	Total
Aggression	2 9 12 14 15 21 24 28 48 49 50 53 54 57 62 65 70 71 77	19
Delinquency	1 3 4 10 11 13 16 17 19 22 23 25 26 29 31 32 33 34 35 36 37 38 39 40 41 42 43 44 45 46 51 55 56 59 60 61 63 64 6667 68 69 72 73 74 76 80 82 84 85 88 92 93 94 96 99 100	57
Hyperactivity	5 6 7 8 18 20 27 30 47 75 78 79 83 87 90 91 97 98	18
Neither	52 58 81 86 89 95	6

## 13 Statistical analysis of the three behavioral constructs

Externalizing behavior was classified into three behavioral constructs, namely; aggression, delinquency, and hyperactivity.

### 13.1 Gender and the trio of aggression, delinquency and hyperactivity

The descriptive statistics presented in Table 14, shows that the male scored higher than the female in the three behaviors. The Mann-Whitney test shows that the mean scores of males and females are the same for aggression and hyperactivity. However, the mean score is different for delinquent behavior.

**Table 14:** Descriptive statistics and t test of gender and the three behaviors.

Behavior	Statistic	Male	Female	W	P-value
Aggression	Mean	17.91	17.63	694240.5	0.406
	Median	18	18		
	St. Dev	7.226	6.719		
	Sum	13862	17563		
	Total	774	996		
Delinquency	Mean	40.69	38.5	857698.5	0.0229
	Median	39	37		
	St. Dev	17.538	15.752		
	Total	31496	38344		
Hyperactivity	Mean	14.49	14.33	876348.5	0.599
	Median	14	14		
	St. Dev	5.963	5.779		
	Sum	11214	14276		

Two-way analysis of variance presented in Table 15, showed that the mean scores of the three behaviors across the gender are different. Similarly, the mean score between the genders across the three behaviors is different. The interaction between the gender and the trio of aggression, delinquency, and hyperactivity is significant.

**Table 15:** ANOVA assessing interaction between gender and the three behaviors.

Source	SS	Df	MS	F
Rows (R)	654964.02	2	327482.01	2754.29***
Column (C)	1000.82	1	1000.82	8.42***
R x C	1140.34	2	570.17	4.8***
Error	630639.45	5304	118.9	
Total	1287744.63	5309		

***p < 0.001.

### 13.2 Age and the trio of aggression, delinquency and hyperactivity

The descriptive statistics presented in Table 16, showed the respondents aged between 11 and 15 scored highest in aggression. Respondents aged 10 and below scored highest in delinquency and hyperactivity. The Kruskal Wallis H testshowed that the mean scores of all the age groups are the same in aggression and delinquency but different in hyperactivity.

**Table 16:** Descriptive statistics and one-way ANOVA of age and the three behaviors.

Behavior	Statistic	≤10	11≤15	16≤20	H-value	P-value
Aggression	Mean	17.29	17.92	17.44	2.353	0.3084
	Median	17	18	18		
	St. Dev	7.952	6.752	7.089		
	Sum	2698	21541	7186		
	Total	156	1202	412		
Delinquency	Mean	40.82	39.41	39.09	1.184	0.5531
	Median	39	38	37		
	St. Dev	17.572	16.616	16.131		
	Sum	6368	47368	16104		
Hyperactivity	Mean	15.35	14.56	13.57	13.703	0.0011
	Median	16	14	13		
	St. Dev	6.134	5.827	5.766		
	Sum	2394	17505	5591		

Two-way analysis of variance presented in Table 17, showed that the mean scores of the three behaviors across the age groups are different. However, the mean score among the age groups across the three behaviors is the same. The interaction between the age and the trio of aggression, delinquency, and hyperactivity is not significant.

**Table 17:** ANOVA assessing interaction between age and the three behaviors.

Source	SS	Df	MS	F
Rows (R)	654964.02	2	327482.01	2747.37***
Column (C)	523.42	2	261.71	2.2
R x C	387.62	4 5301 5309	96.91	0.81
Error	631869.57		119.2	
Total	1287744.63			

***p < 0.001.

### 13.3 School type and the trio of aggression, delinquency and hyperactivity

The descriptive statistics presented in Table 18, showed the respondents in private schools scored higher than those in public schools in the three behaviors. The Mann-Whitney test showed that the mean scores of private and public schools are different in all three behaviors.

**Table 18:** Descriptive statistics and one-way ANOVA of school type and the three behaviors.

Behavior	Value	Private	Public	W	P-value
Aggression	Mean	18.51	17.33	597681	0.0004
	Median	19	17		
	St. Dev	6.973	6.895		
	Sum	11738	19687		
	Total	634	1136		
Delinquency	Mean	42.3	37.87	621419	<0.0001
	Median	41	36		
	St. Dev	16.48	16.443		
	Sum	26816	43024		
Hyperactivity	Mean	15.54	13.76	624024	<0.0001
	Median	16	13		
	St. Dev	5.879	5.753		
	Sum	9855	15635		

Two-way analysis of variance presented in Table 19, showed that the mean scores of the three behaviors across the school type are different. Similarly, the mean score between the school types across the three behaviors is different. The interaction between the school types and the trio of aggression, delinquency, and hyperactivity is significant.

**Table 19:** ANOVA assessing interaction between school type and the three behaviors.

Source	SS	df	MS	F
Rows (R)	654964.02	2	327482.01	2788.25***
Column (C)	7404.01	1	7404.01	63.04***
R x C	2418.45	2	1209.22	10.3***
Error	622958.15	5304	117.45	
Total	1287744.63	5309		

***p < 0.001.

### 13.4 School level and the trio of aggression, delinquency and hyperactivity

Two-way analysis of variance presented in Table 20, showed that the mean scores of the three behaviors across the school level are different. Similarly, the mean score between the school level across the three behaviors is different. The Mann-Whitney test between the school levels and the trio of aggression, delinquency, and hyperactivity is significant.

**Table 20:** Descriptive statistics and one-way ANOVA of school level and the three behaviors.

Behavior	Value	Primary	Secondary	W	P value
Aggression	Mean	16.99	17.96	307899	0.0395
	Median	17	18		
	St. Dev	7.651	6.375		
	Sum	6251	25174		
	Total	368	1402		
Delinquency	Mean	42.66	38.62	358044.5	0.0002
	Median	41	37		
	St. Dev	18.671	15.897		
	Sum	15700	54140		
Hyperactivity	Mean	15.17	14.2	352943.5	0.0019
	Median	15	14		
	St. Dev	6.281	5.739		
	Sum	5581	19909		

Two-way analysis of variance presented in Table 21, showed that the mean scores of the three behaviors across the school levels are different. Similarly, the mean score between the school levels across the three behaviors is different. The interaction between the school types and the trio of aggression, delinquency, and hyperactivity is significant.

**Table 21:** ANOVA assessing interaction between school level and the three behaviors.

Source	SS	df	MS	F
Rows (R)	654964.02	2	327482.01	2768.24***
Column (C)	1588.02	1	1588.02	13.42***
R x C	3731.06	2	1865.53	15.77***
Error	627461.53	5304	118.3	
Total	1287744.63	5309		

***p < 0.001.

## 14 Regression analysis

Regression analysis was done using aggression, delinquency and hyperactivity as the respective dependent variables and the demographic variables as the independent variables.

The regression models as shown in Table 22 are significant despite the low values of both the R square and adjusted R square.

**Table 22:** Regression of the three behaviors against the demographic variable.

Behavior	Constant	School type	Age	Gender	School level	Adjusted R Square	F
Aggression	16.890***	1.126***	-0.213	-0.340	1.131*	0.008	4.785***
Aggression II	16.553***	1.189***			0.978*	0.008	4.785***
Delinquency	40.243***	5.165***	2.352***	-1.994*	-5.309***	0.032	15.586***
Hyperactivity	14.773***	1.702***	-0.239	-0.135	-0.797*	0.024	11.816***
Hyperactivity II	14.519***	1.776***			-0.952***	0.024	11.816***

*p < 0.05; ***p < 0.001, Aggression II = controlling for age and gender, Hyperactivity II = controlling for age and gender.

### 14.1 Aggression

The regression coefficient model indicates that school type and school level contributed significantly to the model while gender and age did not.

Controlling for age and gender, yielded the final regression model that establishes the relationship between aggression and the duo of school type and school level.

### 14.2 Delinquency

The regression coefficient model indicates that all four demographics factors contributed significantly to the model.

### 14.3 Hyperactivity

The regression coefficient model indicates that school type and school level contributed significantly to the model while gender and age did not.

Controlling for age and gender, yielded the final regression model that establishes the relationship between hyperactivity and the duo of school type and school level.

## 15 Correspondence analysis

**Figure 1:**
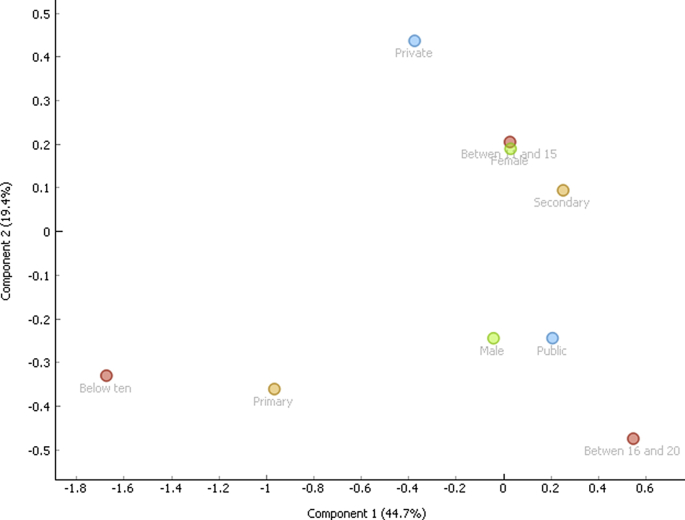
Correspondence plot for aggression and the demographic variables.

**Figure 2:**
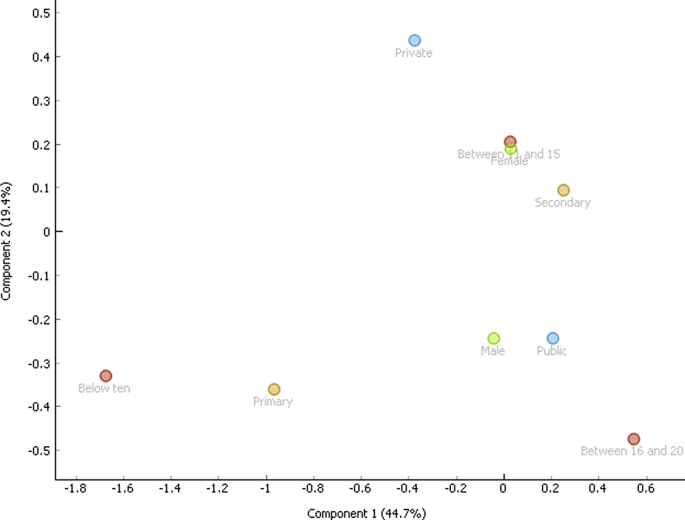
Correspondence plot for delinquency and the demographic variables.

**Figure 3:**
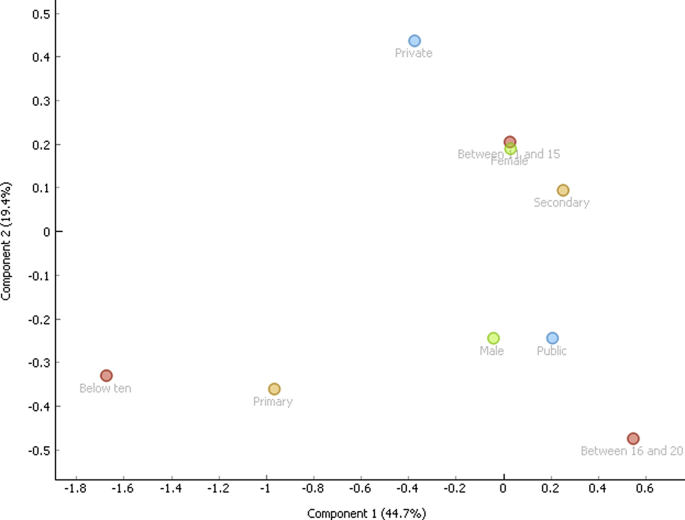
Correspondence plot for hyperactivity and the demographic variables.

Correspondence analysis is a vital tool used to classify variables regardless of the nature of the variables (dependent and independent). The associations are depicted graphically without establishing inferences. The data of the three behaviors were first broken into nine (9) demographic variables namely, public, private, 10 and below, 11-15, 16-20, female, male, primary and secondary respectively. Correspondence analysis was applied and the two-dimensional graphs were obtained for aggression (Figure 1), delinquency (Figure 2) and hyperactivity (Figure 3). In all the instances, the model was able to explain 65% of the variability of the data. Three distinct behavioral patterns were obtained.

i). below 10 and primary
ii). Male, public and between 16 and 20
iii). Private, secondary, female and between 11 and 15.

## 16 Discussion

### 16.1 Externalizing behavior is associated with school type

This work has shown that the externalizing behavior of children and young adolescents is associated with school variety (private and public schools). Furthermore, the association confers different behavioral patterns in privately owned and publicly funded primary and secondary schools. Although, it has been shown by [121] that students in secondary schools have twice more odds to exhibit externalizing behavior than their colleagues in private schools, this present study considered both primary and secondary schools. The outcome is highly expected because of the income disparity in Nigeria. Children from high-income families attend private schools while those from low-income attend public schools [122]. Privately funded schools have a low student to teacher ratio compared with public schools [123], hence, teachers are in a better position to monitor and correct behavior lapses exhibited by the students or pupils. On the other hand, public-funded schools are overcrowded because the population is skewed towards low-income families, and the teachers have enormous workloads and cannot effectively monitor the behaviors of the children. In this case, the emphasis of the teachers is exclusively teaching and not behavioral corrections. The inability of teachers in public schools to adequately monitor the students results to absenteeism [124] and truancy [125], which are some of the manifestations of externalizing behaviors. The low motivation of public school teachers is also a contributory factor.

### 16.2 Externalizing behavior is not associated with age

The present study has shown that externalizing behavior is not associated with age, an indication that externalizing behavioral pattern is the same for ages considered in this work. The implication is that an intervention program must target all the age groups, although some specific age groups may be tagged the riskiest. Early findings indicated that antisocial behavior attenuates as children migrate to adulthood [43]. This is increasingly been disputed since externalizing behavior is a predictor of crimes, violence and substance abuse in adults [38]. This is been reechoed in the present study that externalizing behavioral patterns is the same in children and early adolescents. Moreover, recent studies employ age as a moderating or mediating variable between their studied variables and externalizing behavior. Intimate sexual violence [126] and exposure to greenspaces [127] are examples of the studied variables.

### 16.3 Externalizing behavior is associated with gender

Most studies link externalizing behavior to gender [128]. Hitherto, boys lead girls in any given methodology such as self, teacher scores and parental scores [121]. The present study has extended gender differentials in externalizing behavior disorders to different age groups, school type, and school level.

### 16.4 Externalizing behavior is associated with school level

Externalizing behavior in this study is associated with the school level (primary, secondary) [129]. The findings are opposite to [130] where the study population was restricted to girls only. Surprisingly, this is in variance with age because age is what often determines primary (elementary) and secondary (high school) educational level. The present study has shown that there are hidden variables that confer different externalizing behaviors between primary and secondary schools. Two of the reasons are gender and school types. The respondents externalizing behavior is associated with gender and school type, which spreads across the primary and secondary schools. Primary school pupils that attend private schools are most likely to have different externalizing behavior with those that attend public primary schools. The same applies to secondary schools. Similarly, boys display more externalizing behavior than girls at both primary and secondary schools.

### 16.5 Externalizing behavior differences and the demographics

Contingency analysis showed that the externalizing behavioral patterns differ mostly in school level (87/100), age (63/100), school type (62/100) and gender (37/100) in the given 100 externalizing variables in the questionnaire. This study has shown that the externalizing behavioral pattern is completely different in secondary and primary schools. To fully comprehend the contingency and mean results, the 100 questions in the questionnaires were split into the aggression, delinquency, and hyperactivity. Analysis of the behaviors in bits will reveal some patterns, inadvertently concealed in the whole analysis.

### 16.6 Gender and the trio of aggression, delinquency and hyperactivity

Expectedly, males scored higher than females in aggression, delinquency, and hyperactivity. The present study corroborates the findings of [65] and [67]. Genetic and environmental factors are the prime contributory factors [63]. The present study has shown that delinquent behavior is inclined to boys than girls, which is a submission of [90] and contrary to [88, 89]. The same was observed for hyperactivity [106].

Similar aggressive and hyperactive behavioral patterns were observed for both males and females which is contrary to the findings of [131]. The difference is because of the cumulative effects of demographics used in [131] while the present study is from single demography.

However, delinquent behavioral pattern is different for both genders, although gender differences in delinquent behavior are often moderated by other variables such as incarceration [132] and parenting methods [133]. This research is one of the few that reported similar aggressive and hyperactive behavioral patterns for male and female children and young adolescents.

The interaction between the gender and the trio of aggression, delinquency, and hyperactivity (ADH) is significant. This is an indication that the effects on gender on the three behaviors are different. The finding is parallel to [134], although the authors included resilience, somatic symptoms to aggression, delinquency, and hyperactivity.

### 16.7 Age and the trio of aggression, delinquency and hyperactivity

The respondents aged between 11 and 15 scored highest in aggression. This is expected because that age bracket marks the onset of puberty where hormonal changes can trigger aggressive behaviors [135] such as bullying [136]. The aggression slowed down between ages 16 and 20 which is expected to attenuate or remain latent as the adolescents advance towards adulthood [137].

The present study showed that delinquency decreases as age increases [138]. Ironically, the Kruskal Wallis test showed that age is not related to delinquency. Numerous findings point to the contrary [139, 140]. This is traceable to the fact that most of the studies considered homogenous populations but the present finding considered a heterogeneous one.

Similarly, hyperactivity decreases as the ages of the respondents' increases but a significant association was established between age and hyperactivity. Expectedly, it has been shown that hyperactivity decreases as children migrate to adulthood [141]. The study population and other variables determine whether hyperactivity is associated with age [115, 142, 143].

The interaction between the age and the trio of aggression, delinquency, and hyperactivity is not significant. This explains the reasons why different results are obtained by different researchers and a pointer that a significant interaction is possible if the median is used instead of the mean, the same number of variables for the three behaviors or environmental and biological factors that cannot be captured using questionnaires.

### 16.8 School type and the trio of aggression, delinquency and hyperactivity

This is one of the four areas; this work makes substantial additions to the literature. Unexpectedly, the private schools irrespective of age, gender and level scored higher than the public school in aggression, delinquency, and hyperactivity and their mean scores are different. In the aggression aspect, the present study corroborates the findings of [144], which was a study conducted in the southern Philippines. Similar results on emotional problems have been reported [145]. Caution is advocated in terms of hyperactivity because private schools are often victims of false positives [146].

Although the sample size of public schools is higher than the private schools, the effects are equaled by the average or the mean. Another explanation could be that the teachers in the public schools did not monitor the students/pupils effectively during the period of questionnaire administration or the students in the private schools understand the questionnaire better than those in the public schools [147]. As mentioned earlier, the workload of teachers in public schools could be culpable [148]. The present work has shown the presence of behavioral differences between private and public schools in Nigeria. Nevertheless, this finding has shown that intervention programs should be targeted at private schools if the reduction of the prevalence of behavioral disorders in Nigeria is anticipated. Despite the perceived advantages of private education [149, 150], parents are to be aware that sending their wards there would not shield them from externalizing behavior unless a targeted action is taken to address disruptive behavior observed in them. Even religious private schools are not exceptions [151]. Emphasis should not be limited to quality education and civil responsibilities, ethics and guidance and counseling should be incorporated into their curriculum [152]. Psychiatric evaluation units should be established to manage behavioral profiles, coordinate behavioral corrections, treatments and effectively handle cases of episodes [153]. Research activities are expected to flow towards this area to fully study the behavioral differences between private and public schools in the nursery, primary, secondary, vocational and tertiary levels of education.

The interaction between the school types and the trio of aggression, delinquency, and hyperactivity is significant, an evidence that the three behaviors differ significantly in private and public schools.

### 16.9 School level and the trio of aggression, delinquency and hyperactivity

The second major contribution of the present study presented that aggression is higher in secondary schools, while delinquency and hyperactivity are more prevalent in primary schools. It is an age-long view that aggressors are usually older while their victims are younger [154] and hence, younger people are expected to score lower in aggression scale, as it was the case of primary school pupils [155]. A comparison between aggressiveness in primary and secondary schools is necessary for the design and implementation of intervention programs.

The higher score obtained from primary school pupils corroborates the findings of [156] which stated that hyperactivity is most likely to be prevalent and diagnosed in young children. Hence, the effects of hyperactivity decrease towards adulthood. Similar findings have shown that hyperactive and delinquency behavior attenuates or in this aspect, decreases towards adulthood [157]. Since, hyperactive and delinquent behaviors are diagnosed early (in this case, in the primary schools), intervention methods are highly recommended to address the behavioral disorders before they snowballed into adolescence and possibly adulthood [158, 159, 160, 161].

The interaction between the school level and the trio of aggression, delinquency, and hyperactivity is significant. Since the three behaviors are components of externalizing behavior, it implies that externalizing behavioral pattern is different for primary and secondary schools.

### 16.10 Regression of the behaviors with the demographic variables

The third major contribution of this work is that Regression analysis was used to establish an association between three behaviors and the demographic variables. Firstly, it was discovered that aggression and hyperactivity could be predicted by school type and level after controlling for the duo of age and gender, which contributed infinitesimally to the respective model. Lastly, delinquency can be predicted by age, gender, and school type and school level. All the demographic variables contributed significantly to the model. The present study has shown that school level and type are the strongest predictors of externalizing behavior. Intervention program, especially in this demographic, should consider this in addressing behavioral disorders in the schools, the ages, and gender of the students/pupils notwithstanding.

### 16.11 Exploratory analysis

Correspondence analysis showed a similar behavioral pattern for the three behaviors. This is the last major contribution of this work. Individuals grouped based on the three behavioral clusters can be targeted for intervention.

## Declarations

### Author contribution statement

H.I. Okagbue: Conceived and designed the experiments; Performed the experiments; Analyzed and interpreted the data; Wrote the paper.

S.A. Bishop, J.A. Odukoya: Conceived and designed the experiments; Performed the experiments; Analyzed and interpreted the data.

### Funding statement

This work was supported by the Centre for Research, Innovation and Discovery (CUCRID) at Covenant University.

### Competing interest statement

The authors declare no conflict of interest.

### Additional information

No additional information is available for this paper.

## References

[1] Bishop S.A., Owoloko E.A., Okagbue H.I., Oguntunde P.E., Odetunmibi O.A., Opanuga A.A. (2017). Survey datasets on the externalizing behaviors of primary school pupils and secondary school students in some selected schools in Ogun State, Nigeria. Data in Brief.

[2] Campbell S.B., Shaw D.S., Gilliom M. (2000). Early externalizing behavior problems: toddlers and preschoolers at risk for later maladjustment. Dev. Psychopathol..

[3] Campos R.C., Besser A., Morgado C., Blatt S.J. (2014). Self Criticism, dependency, and adolescents’ externalizing and internalizing problems. Clin. Psychol..

[4] Aldao A., Nolen-Hoeksema S., Schweizer S. (2010). Emotion-regulation strategies across psychopathology: a meta-analytic review. Clin. Psychol. Rev..

[5] Mohamed A.H.H. (2018). Gender as a moderator of the association between teacher–child relationship and social skills in preschool. Early Child. Dev. Care.

[6] Pakarinen E., Silinskas G., Hamre B.K., Metsäpelto R.-L., Lerkkanen M.-K., Poikkeus A.-M., Nurmi J.-E. (2018). Cross-Lagged associations between problem behaviors and teacher-student relationships in early adolescence. J. Early Adolesc..

[7] Bhutta A.T., Cleves M.A., Casey P.H., Cradock M.M., Anand K.J.S. (2002). Cognitive and behavioral outcomes of school-aged children who were born preterm: a meta-analysis. J. Am. Med. Assoc..

[8] Jackson-Browne M.S., Papandonatos G.D., Chen A., Yolton K., Lanphear B.P., Braun J.M. (2019). Early-life triclosan exposure and parent-reported behavior problems in 8-year-old children. Environ. Int..

[9] Min M.O., Minnes S., Park H., Ridenour T., Kim J.-Y., Yoon M., Singer L.T. (2018). Developmental trajectories of externalizing behavior from ages 4 to 12: prenatal cocaine exposure and adolescent correlates. Drug Alcohol Depend..

[10] MacKinnon N., Kingsbury M., Mahedy L., Evans J., Colman I. (2018). The association between prenatal stress and externalizing symptoms in childhood: evidence from the avon longitudinal study of parents and children. Biol. Psychiatr..

[11] Arsenault L., Tremblay R.E., Boulerice B., Saucier J.F. (2002). Obstetrical complications and violent delinquency: testing two developmental pathways. Child Dev..

[12] Kendler K.S., Prescott C., Myers J., Neale M.C. (2003). The structure of genetic and environmental risk factors for common psychiatric and substance use disorders in men and women. Arch. Gen. Psychiatr..

[13] Krueger R.F., Hicks B.M., Patrick C.J., Carlson S.R., Iacono W.G., McGue M. (2002). Etiologic connections among substance dependence, antisocial behavior, and personality: modeling the externalizing spectrum. J. Abnorm. Psychol..

[14] Rodrigues J.L.G., Araújo C.F.S., dos Santos N.R., Bandeira M.J., Anjos A.L.S., Carvalho C.F., Lima C.S., Abreu J.N.S., Mergler D., Menezes-Filho J.A. (2018). Airborne manganese exposure and neurobehavior in school-aged children living near a ferro-manganese alloy plant. Environ. Res..

[15] Lim J., Kweon K., Kim H.-W., Cho S.W., Park J., Sim C.S. (2018). Negative impact of noise and noise sensitivity on mental health in childhood. Noise Health.

[16] Liao T.L., Chen Y.S., Chen C.Y., Chien L.Y. (2014). Self-reported internalizing and externalizing behaviours among junior high school students at 2 and 4 years after the 921 Earthquake in Taiwan. Stress Health.

[17] Lowe S.R., Godoy L., Rhodes J.E., Carter A.S. (2013). Predicting mothers’ report of children’s mental health three years after Hurricane Katrina. J. Appl. Dev. Psychol..

[18] Comeau J., Boyle M.H. (2018). Patterns of poverty exposure and children’s trajectories of externalizing and internalizing behaviors. SSM Populat. Health.

[19] Cheung R.Y.M., Leung M.C., Chung K.K.H., Cheung H.Y. (2018). Family risks and adolescent adjustment in Chinese contexts: testing the mediating role of emotional intelligence. J. Child Fam. Stud..

[20] de Vries E.E., Verlinden M., Rijlaarsdam J., Jaddoe V.W.V., Verhulst F.C., Arseneault L., Tiemeier H. (2018). Like father, like child: early life family adversity and children’s bullying behaviors in elementary school. J. Abnorm. Child Psychol..

[21] Salimiha A., Perales F., Baxter J. (2018). Maternal employment and children’s socio-emotional outcomes: an Australian longitudinal study. Int. J. Publ. Health.

[22] Green V.R., Conway K.P., Silveira M.L., Kasza K.A., Cohn A., Cummings K.M., Stanton C.A., Callahan-Lyon P., Slavit W., Sargent J.D., Hilmi N., Niaura R.S., Reissig C.J., Lambert E., Zandberg I., Brunette M.F., Tanski S.E., Borek N., Hyland A.J., Compton W.M. (2018). Mental health problems and onset of tobacco use among 12- to 24-year-olds in the PATH study. J. Am. Acad. Child Adolesc. Psychiatr..

[23] Mares M.L., Stephenson L., Martins N., Nathanson A.I. (2018). A house divided: parental disparity and conflict over media rules predict children's outcomes. Comput. Hum. Behav..

[24] Goodman S.H., Rouse M.H., Connell A.M., Broth M.R., Hall C.M., Heyward D. (2011). Maternal depression and child psychopathology: a meta-analytic review. Clin. Child Fam. Psychol. Rev..

[25] Rolan E., Marceau K. (2018). Individual and sibling characteristics: parental differential treatment and adolescent externalizing behaviors. J. Youth Adolesc..

[26] Godleski S.A., Crane C.A., Leonard K.E. (2018). Parents' concordant and discordant alcohol use and subsequent child behavioral outcomes. Addict. Behav..

[27] Conway L.J., Levickis P.A., Mensah F., McKean C., Smith K., Reilly S. (2017). Associations between expressive and receptive language and internalizing and externalizing behaviours in a community-based prospective study of slow-to-talk toddlers. Int. J. Lang. Commun. Disord..

[28] Elbedour S., Onwuegbuzie A.J., Alatamin M. (2003). Behavioral problems and scholastic adjustment among Bedouin-Arab Children from polygamous and monogamous marital family structures: some developmental considerations. Genet. Soc. Gen. Psychol. Monogr..

[29] Cooke M.E., Neale Z.E., Barr P.B., Myers J., Dick D.M., Kendler K.S., Edwards A.C. (2017). The role of social, familial, and individual-level factors on multiple alcohol use outcomes during the first year of university. Alcohol Clin. Exp. Res..

[30] Ekinci O., Topcuoglu V., Sabuncuoglu O., Berkem M., Akin E., Gumustas F.O. (2012). The association of tattooing, body piercing and psychopathology in adolescents: a community based study from Istanbul. Community Ment. Health J..

[31] Ruhland E.L., Davis L., Atella J., Shlafer R.J. (2020). Externalizing behavior among youth with a current or formerly incarcerated parent. Int. J. Offender Ther. Comp. Criminol..

[32] Ingul J.M., Klockner C.A., Silverman W.K., Nordahl H.M. (2012). Adolescent school absenteeism: modeling social and individual risk factors. Child Adolesc. Ment. Health.

[33] Nik Jaafar N.R., Tuti Iryani M.D., Wan Salwina W.I., Fairuz Nazri A.R., Kamal N.A., Prakash R.J., Shah S.A. (2013). Externalizing and internalizing syndromes in relation to school truancy among adolescents in high-risk urban schools. Asia Pac. Psychiatr..

[34] Kubiszewski V., Fontaine R., Huré K., Rusch E. (2013). Cyber-bullying in adolescents: associated psychosocial problems and comparison with school bullying. Encephale.

[35] Kerebih H., Abrha H., Frank R., Abera M. (2018). Perception of primary school teachers to school children's mental health problems in Southwest Ethiopia. Int. J. Adolesc. Med. Health.

[36] Perry K.J., Price J.M. (2018). Concurrent child history and contextual predictors of children's internalizing and externalizing behavior problems in foster care. Child. Youth Serv. Rev..

[37] Proctor L.J., Lewis T., Roesch S., Thompson R., Litrownik A.J., English D., Arria A.M., Isbell P., Dubowitz H. (2017). Child maltreatment and age of alcohol and marijuana initiation in high-risk youth. Addict. Behav..

[38] Low S., Tiberio S.S., Shortt J.W., Mulford C., Eddy J.M., Capaldi D.M. (2017). Intergenerational transmission of violence: the mediating role of adolescent psychopathology symptoms. Dev. Psychopathol..

[39] Ueda M. (2017). Developmental risk factors of juvenile sex offenders by victim age: an implication for specialized treatment programs. Aggress. Violent Behav..

[40] Hinshaw S.P. (1987). On the distinction between attentional deficits/hyperactivity and conduct problems/aggression in child psychopathology. Psychol. Bull..

[41] Hinshaw S.P. (1992). Externalizing behavior problems and academic underachievement in childhood and adolescence: casual relationships and underlying mechanisms. Psychol. Bull..

[42] Duncan G.J., Dowsett C.J., Claessens A., Magnuson K., Huston A.C., Klebanov P., Pagani L.S., Feinstein L., Engel M., Brooks-Gunn J., Sexton H., Duckworth K., Japel C. (2007). School readiness and later achievement. Dev. Psychol..

[43] Liu J. (2004). Childhood externalizing behavior: theory and implications. J. Child Adolesc. Psychiatr. Nurs..

[44] Berke D.S., Reidy D., Zeichner A. (2018). Masculinity, emotion regulation, and psychopathology: a critical review and integrated model. Clin. Psychol. Rev..

[45] Lin Y.-C., Lin C.-Y. (2018). Minor symptoms talk: how children react to encountered bullying. Child Indicat. Res..

[46] Jerome E.M., Hamre B.K., Pianta R.C. (2009). Teacher-child relationships from kindergarten to sixth grade: early childhood predictors of teacher-perceived conflict and closeness. Soc. Dev..

[47] Raval G., Montañez E., Meyer D., Berger-Jenkins E. (2019). School-based mental health promotion and prevention program “turn 2 Us” reduces mental health risk behaviors in urban, minority youth. J. Sch. Health.

[48] Demirtaş-Zorbaz S., Ergene T. (2019). School adjustment of first-grade primary school students: effects of family involvement, externalizing behavior, teacher and peer relations. Child. Youth Serv. Rev..

[49] Ansary N.S., Luthar S.S. (2009). Distress and academic achievement among adolescents of affluence: a study of behaviors and school performance. Dev. Psychopathol..

[50] Palmu I.R., Närhi V.M., Savolainen H.K. (2018). Externalizing behaviour and academic performance–the cross-lagged relationship during school transition. Emot. Behav. Difficult..

[51] McCoy D.C., Jones S., Roy A., Raver C.C. (2018). Classifying trajectories of social-emotional difficulties through elementary school: impacts of the Chicago school readiness project. Dev. Psychol..

[52] Zimmermann F., Schuttle K., Taskinen P., Koller O. (2013). Reciprocal effects between adolescent externalizing problems and measures of achievement. J. Educ. Psychol..

[53] Loginova S.V. (2018). Parenting as a mediator in the relation between child individual differences and externalizing behavior. Psikhologicheskii Zhurnal.

[54] Na K.S., Lee S.I., Hong H.J., Oh M.J., Bahn G.H., Ha K., Shin Y.M., Song J., Park E.J., Yoo H., Kim H., Kyung Y.M. (2014). The influence of unsupervised time on elementary school children at high risk for inattention and problem behaviors. Child Abuse Negl..

[55] Moeijes J., Van Busschbach J.T., Bosscher R.J., Twisk J.W.R. (2018). Sports participation and psychosocial health: a longitudinal observational study in children. BMC Publ. Health.

[56] Benner G.J., Ron Nelson J., Sanders E.A., Ralston N.C. (2012). Behavior intervention for students with externalizing behavior problems: primary-level standard protocol. Except. Child..

[57] Lyons M.D., Otis K.L., Huebner E.S., Hills K.J. (2014). Life satisfaction and maladaptive behaviors in early adolescents. Sch. Psychol. Q..

[58] Farrington D.P. (1989). Early predictors of adolescent aggression and adult violence. Violence Vict..

[59] Shaw D.S., Giliom M., Giovannelli J., Zeanah C.H. (2000). Aggressive behavior disorders.

[60] Reebye P.N. (2005). Aggression during early years-infancy and preschool. J. Canad. Acad. Child Adolesc. Psychiatry.

[61] Galal Y.S., Emadeldin M., Mwafy M.A. (2019). Prevalence and correlates of bullying and victimization among school students in rural Egypt. J. Egypt. Publ. Health Assoc..

[62] Farrington D.P., Pinard G.F., Pagani L. (2001). Predicting adult official and self-reported violence. Clinical Assessment of Dangerousness: Empirical Contributions.

[63] Carroll A., McCarthy M., Houghton S., Sanders O'Connor E., Zadow C. (2018). Reactive and proactive aggression as meaningful distinctions at the variable and person level in primary school-aged children. Aggress. Behav..

[64] Drury A.J., Elbert M.J., DeLisi M. (2019). Childhood sexual abuse is significantly associated with subsequent sexual offending: new evidence among federal correctional clients. Child Abuse Negl..

[65] Petot D., Grandjean P., Petot J.-M., Gonthier V., Chalvidan E. (2017). Screening and analysing the harassment at school: the PPC-17 self-report questionnaire. Psychol. Fr..

[66] Hadley M. (2003). Relational, indirect, adaptive, or just mean: recent work on aggression in adolescent girls—Part I. Stud. Gend. Sex..

[67] Card N.A., Stucky B.D., Sawalani G.M., Little T.D. (2008). Direct and indirect aggression during childhood and adolescence: a meta-analytic review of gender differences, intercorrelations, and relations to maladjustment. Child Dev..

[68] Chan S.F., La Greca A.M., Peugh J.L. (2019). Cyber victimization, cyber aggression, and adolescent alcohol use: short-term prospective and reciprocal associations. J. Adolesc..

[69] Lei H., Chiu M.M., Cui Y., Zhou W., Li S. (2018). Parenting style and aggression: a meta-analysis of mainland Chinese children and youth. Child. Youth Serv. Rev..

[70] Wang M. (2019). Harsh parenting and adolescent aggression: adolescents’ effortful control as the mediator and parental warmth as the moderator. Child Abuse Negl..

[71] Beckmann L., Bergmann M.C., Fischer F., Mößle T. (2017). Risk and protective factors of child-to-parent violence: a comparison between physical and verbal aggression. J. Interpers. Viol..

[72] Tieskens J.M., Buil J.M., Koot S., Krabbendam L., van Lier P.A.C. (2018). Elementary school children's associations of antisocial behaviour with risk-taking across 7–11 years. J. Child Psychol. Psychiatry Allied Discip..

[73] Dodo O., Muzenje A. (2019). Violence in schools in seke district: influences and implications for social policy. Afr. J. Soc. Work.

[74] Carraro A., Gobbi E. (2018). Play fighting to cope with children aggression: a study in primary school. J. Phys. Educ. Sport.

[75] Siegel L.J., Welsh B. (2011). Juvenile Delinquency: the Core.

[76] Achembach T.M., Edelbrock C.S. (1983). Manual for Child Behavior Checklist and Revised Child Behavior Profile.

[77] Steinberg L. (2008). Adolescence.

[78] Gentina E., Tang T.L.-P., Gu Q. (2017). Does bad company corrupt good morals? Social bonding and academic cheating among French and Chinese teens. J. Bus. Ethics.

[79] Bax T., Hlasny V. (2019). The causes and courses of nonviolent and violent delinquency among south Korean adolescents. Deviant Behav..

[80] Bartol C., Bartol A. (2009). Juvenile Delinquency and Antisocial Behavior: A Developmental Perspective.

[81] Zavala E., Spohn R.E., Alarid L.F. (2019). Gender and serious youth victimization: assessing the generality of self-control, differential association, and social bonding theories. Socio. Spectr..

[82] Turner R., Daneback K., Skårner A. (2018). Assessing reciprocal association between drunkenness, drug use, and delinquency during adolescence: separating within- and between-person effects. Drug Alcohol Depend..

[83] Popa I., Borrelli P., Breda-Popa R., Montomoli C. (2018). Juvenile delinquency in Romania: a comparison between minors in prisons and re-education services. Eur. J. Soc. Work.

[84] Sarý E., Arslantaþ H. (2019). The attitudes of high school adolescent toward crime and risk factors. Klinik Psikiyatri Dergisi.

[85] Voisin D.R., Sales J.M., Hong J.S., Jackson J.M., Rose E.S., DiClemente R.J. (2017). Social context and problem factors among youth with juvenile justice involvement histories. Behav. Med..

[86] Janssen H.J., Eichelsheim V.I., Deković M., Bruinsma G.J.N. (2017). Sex differences in longitudinal pathways from parenting to delinquency. Eur. J. Crim. Pol. Res..

[87] Yoo J.A. (2017). Effect of child gender on the bidirectional relationships between parental monitoring and delinquent behavior. J. Child Fam. Stud..

[88] Habersaat S., Suter M., Stephan P., Urben S. (2018). Contribution of implicit/explicit self-esteem and gender in psychopathic traits at adolescence. Crim. Justice Behav..

[89] Liu R.X. (2019). Relational strains and delinquency: assessing the gendering of emotions’ claims among Chinese adolescents. Socio. Inq..

[90] Müller C.M., Hofmann V., Arm S. (2017). Susceptibility to classmates’ influence on delinquency during early adolescence. J. Early Adolesc..

[91] Ayer L., Setodji C., Schultz D., Jaycox L.H., Kofner A. (2017). Change in externalizing problems over time among ethnic minority youth exposed to violence. Child. Youth Serv. Rev..

[92] Mercer N., Crocetti E., Branje S., van Lier P., Meeus W. (2017). Linking delinquency and personal identity formation across adolescence: examining between- and within-person associations. Dev. Psychol..

[93] Becerra L.A.A., Serra J.C. (2019). Impact of partner violence on female delinquency. Soc. Sci..

[94] Morgan P.L., Farkas G., Hillemeier M.M., Wang Y., Mandel Z., DeJarnett C., Maczuga S. (2019). Are students with disabilities suspended more frequently than otherwise similar students without disabilities?. J. Sch. Psychol..

[95] Vaughn M.G., Oh S., Salas-Wright C.P., DeLisi M., Holzer K.J., McGuire D. (2019). Sex differences in the prevalence and correlates of handgun carrying among adolescents in the United States. Youth Violence Juv. Justice.

[96] Habersaat S., Boonmann C., Schmeck K., Stéphan P., Francescotti E., Fegert J.M., Perler C., Schmid M., Urben S. (2019). Gender differences in the relationship between strain, negative emotions and delinquent behaviors in institutionalized juveniles. Deviant Behav..

[97] Espelage D.L., Merrin G.J., Hong J.S., Resko S.M. (2018). Applying social cognitive theory to explore relational aggression across early adolescence: a within- and between-person analysis. J. Youth Adolesc..

[98] American Psychiatric Association. 4th ed. Washington, DC: Author (1994). Diagnostic and Statistical Manual of Mental Disorders.

[99] Morrill M.S. (2018). Special education financing and ADHD medications: a bitter pill to swallow. J. Pol. Anal. Manag..

[100] Tao J., Jiang X., Wang X., Liu H., Qian A., Yang C., Chen H., Li J., Ye Q., Wang J., Wang M. (2017). Disrupted control-related functional brain networks in drug-naive children with attention-deficit/hyperactivity disorder. Front. Psychiatr..

[101] Chen H., Song Y., Li X. (2019). A deep learning framework for identifying children with ADHD using an EEG-based brain network. Neurocomputing.

[102] Cowell W.J., Bellinger D.C., Wright R.O., Wright R.J. (2019). Antenatal active maternal asthma and other atopic disorders is associated with ADHD behaviors among school-aged children. Brain Behav. Immun..

[103] Dalsager L., Fage-Larsen B., Bilenberg N., Jensen T.K., Nielsen F., Kyhl H.B., Grandjean P., Andersen H.R. (2019). Maternal urinary concentrations of pyrethroid and chlorpyrifos metabolites and attention deficit hyperactivity disorder (ADHD) symptoms in 2-4-year-old children from the Odense Child Cohort. Environ. Res..

[104] Eckrich S.J., Rapport M.D., Calub C.A., Friedman L.M. (2019). Written expression in boys with ADHD: the mediating roles of working memory and oral expression. Child Neuropsychol..

[105] Sergeant J. (2000). The cognitive-energetic model: an empirical approach to Attention-Deficit Hyperactivity Disorder. Neurosci. Biobehav. Rev..

[106] May T., Adesina I., McGillivray J., Rinehart N.J. (2019). Sex differences in neurodevelopmental disorders. Curr. Opin. Neurol..

[107] Öner Ö., Vatanartıran S., Karadeniz Ş. (2019). Grade effects on teacher ratings of ADHD symptoms among primary school students. Scand. J. Psychol..

[108] Pardini D.A., Byrd A.L., Hawes S.W., Docherty M. (2018). Unique dispositional precursors to early onset conduct problems and criminal offending in adults. J. Am. Acad. Child Adolesc. Psychiatr..

[109] Ulzen T.P., Higginbotham J.C., Donnir G., Jerome L., Segal A. (2018). Undiagnosed attention deficit/hyperactivity disorder (ADHD) among unionized drivers in Ghana: public health and policy implications. Accid. Anal. Prev..

[110] Wymbs B.T., Dawson A.E., Suhr J.A., Bunford N., Gidycz C.A. (2017). ADHD Symptoms as risk factors for intimate partner violence perpetration and victimization. J. Interpers Violence.

[111] Biondic D., Wiener J., Martinussen R. (2019). Parental psychopathology and parenting stress in parents of adolescents with attention-deficit hyperactivity disorder. J. Child Family Issues.

[112] Li Y., Yang J.-W., Zhou Y.-Q., Cao J.-Q., Yang J., Jia X.-R. (2017). Analysis on the problem behaviors and influencing factors in children with attention-deficit hyperactivity disorder. Chin. Gen. Prac..

[113] Johnson S.E., Lawrence D., Perales F., Baxter, Zubrick S.R. (2019). Poverty, parental mental health and child/adolescent mental disorders: findings from a national Australian survey. Child Indicat. Res..

[114] Dir A.L., Hummer T.A., Aalsma M.C., Hulvershorn L.A. (2019). Pubertal influences on neural activation during risky decision making in youth with ADHD and disruptive behavior disorders. Develop. Cogn. Neurosci..

[115] Mowlem F.D., Agnew-Blais J., Pingault J.-B., Asherson P. (2019). Evaluating a scale of excessive mind wandering among males and females with and without attention-deficit/hyperactivity disorder from a population sample. Sci. Rep..

[116] Jiménez-Figueroa G., Ardila-Duarte C., Pineda D.A., Acosta-López J.E., Cervantes-Henríquez M.L., Pineda-Alhucema W., Cervantes-Gutiérrez J., Quintero-Ibarra M., Sánchez-Rojas M., Vélez J.I., Puentes-Rozo P.J. (2017). Prepotent response inhibition and reaction times in children with attention deficit/hyperactivity disorder from a Caribbean community. ADHD Attent. Def. Hyperact. Disord..

[117] Ahn J.-S., Min S., Kim M.-H. (2017). The role of uncontrolled eating and screen time in the link of attention deficit hyperactivity disorder with weight in late childhood. Psychiatry Invest..

[118] Kim K.M., Lim M.H., Kwon H.-J., Yoo S.-J., Kim E.-J., Kim J.W., Ha M., Paik K.C. (2018). Associations between attention-deficit/hyperactivity disorder symptoms and dietary habits in elementary school children. Appetite.

[119] Farhangi M.A., Dehghan P., Jahangiry L. (2018). Mental health problems in relation to eating behavior patterns, nutrient intakes and health related quality of life among Iranian female adolescents. PLoS One.

[120] Suchert V., Pedersen A., Hanewinkel R., Isensee B. (2017). Relationship between attention-deficit/hyperactivity disorder and sedentary behavior in adolescence: a cross-sectional study. ADHD Attent. Def. Hyperact. Disord..

[121] Sharma B., Rai M.K., Sharma A., Karki S. (2019). Emotional and behavioal problems among adolescents in pokhara city in Nepal. J. Nepal Health Res. Counc..

[122] Härmä J. (2013). Access or quality? Why do families living in slums choose low-cost private schools in Lagos, Nigeria?. Oxf. Rev. Educ..

[123] Opanuga A.A., Okagbue H.I., Oguntunde P.E., Bishop S.A., Ogundile O.P. (2019). Learning analytics: issues on the pupil-teacher ratio in public primary schools in Nigeria. Int. J. Emerg. Technol. Learn..

[124] Knollmann M., Reissner V., Hebebrand J. (2019). Towards a comprehensive assessment of school absenteeism: development and initial validation of the inventory of school attendance problems. Eur. Child Adolesc. Psychiatr..

[125] Quin D. (2019). Levels of problem behaviours and risk and protective factors in suspended and non-suspended students. Educ. Develop. Psychol..

[126] Fong V.C., Hawes D., Allen J.L. (2019). A Systematic Review of Risk and Protective factors for externalizing problems in children exposed to intimate partner violence. Trauma Violence Abuse.

[127] Madzia J., Ryan P., Yolton K., Percy Z., Newman N., LeMasters G., Brokamp C. (2019). Residential greenspace association with childhood behavioral outcomes. J. Pediatr..

[128] Syed E.U., Hussein S.A., Haidry S.E.Z. (2009). Prevalence of emotional and behavioral problems among primary school children in Karachi, Pakistan-Multi informat survey. Indian J. Pediatr..

[129] Smith G.C., Hayslip B., Webster B.A. (2019). Psychological difficulties among custodial grandchildren. Child. Youth Serv. Rev..

[130] Silinskas G., Kiuru N., Aunola K., Metsapelto R.–L., Lerkkanem M.–K., Nurmi J.E. (2020). Maternal Affection moderates the associations betwenn parenting stress and early adolescents’ externalizing and internalizing behavior. J. Early Adolesc..

[131] Gershon J. (2002). A meta-analytic review of gender differences in ADHD. J. Atten. Disord..

[132] Anderson V.R., Davidson W.S., Barnes A.R., Campbell C.A., Petersen J.L., Onifade E. (2016). The differential predictive validity of the youth level of service/case management inventory: the role of gender. Psychol. Crime Law.

[133] Shoenberger N., Rocheleau G.C. (2017). Effective parenting and self control: difference by gender. Women Crim. Justice.

[134] Zaharakis N.M., Mason M.J., Brown A., Moore M., Garcia C., Foster R., Richards S. (2018). Resiliency moderates the influence of somatization on externalizing problems. J. Child Fam. Stud..

[135] Nguyen T.–V., McCracken J.T., Albaugh M.D., Botteron K.N., Hudziak J.J., Ducharme S. (2016). A testosterone-related structural brain phenotype predicts aggressive behavior from childhood to adulthood. Psychoneuroendocrinology.

[136] Poláková V.B. (2018). Occurrence and understanding of the issues of bullying in primary schools in Banska Bystrica. Univ. J. Educ. Res..

[137] Messina M., Di Sarno A.D., Mirko Alfano Y., Guastaferno M., Nugnes N., Iennaco D., Maldonado N.M., Sperandeo R., Nascivera N. (2018). Aggression or Aggressiveness?: a research hypothesis on aggression, videogames and executive functions in preschool age.

[138] Cho M., Haight W., Choi W.S., Hong S., Piescher K. (2019). A prospective, longitudinal study of risk factors for early onset of delinquency among maltreated youth. Child. Youth Serv. Rev..

[139] Vega-Cauich J.I., Zumárraga-García F.M. (2019). Factors associated with the onset and actual consumption of substances in juvenile offenders. Anu. Psicol. Juríd..

[140] Walters G.D. (2019). Tracing the delinquency acquisition sequence from older siblings, to friends, to self: a mediation analysis. J. Adolesc..

[141] Asherson P., Agnew-Blais J. (2019). Annual Research Review: does late-onset attention-deficit/hyperactivity disorder exist?. J. Child Psychol. Psychiatry Allied Discip..

[142] Ghanizadeh A., Salehi A., Moeini S.R. (2019). Clinical presentation of attention-deficit hyperactivity disorder symptoms in terms of gender and chronological age. Int. J. Commun. Based Nurs. Midwif..

[143] Murray A.L., Booth T., Eisner M., Auyeung B., Murray G., Ribeaud D. (2019). Sex differences in ADHD trajectories across childhood and adolescence. Dev. Sci..

[144] Campano J.P., Munakata T. (2004). Anger and aggression among Filipino students. Adolescence.

[145] Shakir M., Lodhi I.S. (2019). Relationship between emotional-behavioral problems and academic achievement among government and private schools children. Int. J. Innov. Teach. Learn.

[146] Arruda M., Arruda R., Bigal M., Guidetti V. (2019). Disparities in the diagnosis and treatment of attention-deficit/hyperactivity disorder in children and adolescents-a nationwide study. Neurology.

[147] Ajayi I.A., Ekundayo H.T. (2011). Factors determining the effectiveness of secondary schools in Nigeria. Anthropol..

[148] Anomneze E.A., Ugwu D.I., Enwereuzor I.K., Ugwu L.I. (2016). Teachers’ emotional labour and burnout: does perceived organizational support matter?. Asian Soc. Sci..

[149] Baum D.R., Abdul-Hamid H., Wesley H.T. (2018). Inequality of educational opportunity: the relationship between access, affordability, and quality of private schools in Lagos, Nigeria. Oxf. Rev. Educ..

[150] Ogundile O.P., Bishop S.A., Okagbue H.I., Ogunniyi P.O., Olanrewaju A.M. (2019). Factors influencing ICT adoption in some selected secondary schools in Ogun State, Nigeria. Int. J. Emerg. Technol. Learn..

[151] Figlio D., Ludwig J. (2012). Sex, drugs, and catholic schools: private schooling and non-market adolescent behaviors. Ger. Econ. Rev..

[152] Chikaodi O., Abdulmanan Y., Emmanuel A.T., Muhammad J., Mohammed M.A., Izegboya A., Donald O.O., Balarabe S. (2019). Bullying, its effects on attitude towards class attendance and the contribution of physical and dentofacial features among adolescents in Northern Nigeria. Int. J. Adolesc. Med. Health.

[153] Oduguwa A.O., Adedokun B., Omigbodun O.O. (2017). Effect of a mental health training programme on Nigerian school pupils' perceptions of mental illness. Child Adolesc. Psychiatr. Ment. Health.

[154] Aizenkot D., Kashy-Rosenbaum G. (2019). Cyberbullying victimization in WhatsApp classmate groups among Israeli elementary, middle, and high school students. J. Interpers Violence.

[155] Akcan A., Ergun A. (2019). The effect of an aggressive behavior prevention program on kindergarten students. Publ. Health Nurs..

[156] Layton T.J., Barnett M.L., Hicks T.R., Jena A.B. (2018). Attention deficit-hyperactivity disorder and month of school enrollment. N. Engl. J. Med..

[157] Talepasand S., Mohammadi M.R., Alavi S.S., Khaleghi A., Sajedi Z., Akbari P., Lari M., Kasaeian R., Eskandaripour M., Rashti E., Khaneghahi F.Y., Rashidi F., Hosseini S.J. (2019). Psychiatric disorders in children and adolescents: prevalence and sociodemographic correlates in Semnan Province in Iran. Asian J. Psychiatry.

[158] Bishop S.A., Okagbue H.I., Oludayo O.A., Agboola O.O., Agarana M.C., Adamu M.O. (2018). Survey dataset on the types, prevalence and causes of deviant behavior among secondary school adolescents in some selected schools in Benin City, Edo State, Nigeria. Data in Brief.

[159] Heckman J., Pinto R., Savelyev P. (2013). Understanding the mechanisms through which an influential early childhood program boosted adult outcomes. Am. Econ. Rev..

[160] Buchanan-Pascall S., Gray K.M., Gordon M., Melvin G.A. (2018). Systematic review and meta-analysis of parent group interventions for primary school children aged 4-12 years with externalizing and/or internalizing problems. Child Psychiatr. Hum. Dev..

[161] Reinelt T., Samdan G., Kiel N., Petermann F. (2019). Predicting externalizing behavior problems in early childhood: evidence from longitudinal studies. Kindh. Entwickl..

